# The effects of bilateral prostriata lesions on spatial learning and memory in the rat

**DOI:** 10.3389/fnbeh.2022.1010321

**Published:** 2022-11-09

**Authors:** Shun-Yu Zhang, Sheng-Qiang Chen, Jin-Yuan Zhang, Chang-Hui Chen, Xiao-Jun Xiang, Hui-Ru Cai, Song-Lin Ding

**Affiliations:** ^1^Key Laboratory of Neuroscience, School of Basic Medical Sciences, Guangzhou Medical University, Guangzhou, China; ^2^Department of Psychology, School of Health Management, Guangzhou Medical University, Guangzhou, China; ^3^Allen Institute for Brain Science, Seattle, WA, United States

**Keywords:** prostriata, open field test, elevated plus-maze test, Morris water maze test, spatial learning and memory, anxiety, presubiculum

## Abstract

Area prostriata is the primary limbic structure for rapid response to the visual stimuli in the far peripheral visual field. Recent studies have revealed that the prostriata receives inputs not only from the visual and auditory cortices but also from many structures critical for spatial processing and navigation. To gain insight into the functions of the prostriata in spatial learning and memory the present study examines the effects of bilateral lesions of the prostriata on motor ability, exploratory interest and spatial learning and memory using the open field, elevated plus-maze and Morris water maze tests. Our results show that the spatial learning and memory abilities of the rats with bilateral prostriata lesions are significantly reduced compared to the control and sham groups. In addition, the lesion rats are found to be less interested in space exploration and more anxious while the exercise capacity of the rats is not affected based on the first two behavioral tests. These findings suggest that the prostriata plays important roles in spatial learning and memory and may be involved in anxiety as well.

## Introduction

Area prostriata (prostriata, Pro) was described in the brains of non-human primates (NHP) and humans over 50 years ago (Sanides, 1969; Allman and Kaas, 1971; Sousa et al., 1991; Barbas, 1993; Ding et al., 2003). Previous studies on the prostriata were mainly carried out in NHP including marmoset and macaque monkeys. However, the prostriata in the monkeys as well as in humans is located deep into the anterior calcarine fissure (Sanides, 1969; Morecraft et al., 2000; Ding et al., 2003, 2016) and this makes the prostriata difficult to be targeted with precise injections of neural tracers or lesion chemicals. Consequently, there are less data available about the brain-wide connections and functions of the prostriata. Its efferent projections were only found to reach to the primary visual cortex (V1), association auditory cortex (A2), medial orbitofrontal cortex (ORBm), middle temporal area and cingulate motor area in the NHP brains (Sousa et al., 1991; Barbas, 1993; Rosa et al., 1993; Cavada et al., 2000; Morecraft et al., 2000; Falchier et al., 2010; Rockland, 2012) and no afferent projections were reported in NHP.

In 2013, the mouse equivalent of the prostriata was discovered (Ding, 2013). Recently, Ding et al. have also reported the homologous prostriata in the rats and brain-wide connections of the prostriata in both rats and mice (Chen et al., 2020, 2021; Hu et al., 2020; Lu et al., 2020). Briefly, the prostriata in the rats and mice receives its main inputs from the dorsal lateral geniculate nucleus (DLG), primary and secondary visual and auditory cortices and the cortical regions important for spatial processing and navigation such as subiculum (Sub), presubiculum (PrS; including dorsal PrS or postsubiculum; PrSd-PoS), retrosplenial cortex (RS), medial entorhinal cortex (MEC), anterior thalamic nuclei [ATN, including anterodorsal (AD), anteroventral (AV), anteromedial (AM), and laterodorsal (LD) thalamic nuclei] as well as from the contralateral prostriata (Ding, 2013; Chen et al., 2020, 2021; Ding et al., 2020; Hu et al., 2020; Lu et al., 2020). The efferent projections of the prostriata mainly reach to the V1, PrS-PoS and the subcortical regions that is important for visuomotor behaviors such as lateroposterior thalamic nucleus-pulvinar complex (LP-Pul), ventral lateral geniculate nucleus (VLG), pretectal nucleus (PTN), zone incerta (ZI), and pontine nucleus (PN) (Chen et al., 2021).

Previous studies of the NHP and human brains revealed that the prostriata plays an important role in the rapid processing and analysis of information from far peripheral visual field (Yu et al., 2012; Mikellidou et al., 2017; Tamietto and Leopold, 2018). This function is consistent with the strong and direct projections from the rostral DLG and medial V1 (both receiving inputs from peripheral visual fields) to the prostriata (Lu et al., 2020; Chen et al., 2021). However, our recent studies have also revealed moderate prostriata connections with auditory and olfactory cortices as well as strong connections with spatial memory system structures such as the Sub, PrS, RS, MEC, and ATN. These findings indicate that the prostriata may also play critical roles in multimodal sensory integration and spatial learning and memory. In addition, the prostriata strongly projects to the LP-Pul, which has strong projections to the amygdale, a critical structure for emotion and anxiety. Based on these findings, the first aim of the present study is to chemically damage bilateral prostriata in the rats and examine the effects of these lesions on spatial learning and memory ability. Our second aim is to study the effects of the prostriata lesions on the rat’s anxiety behaviors. Our additional aim is to explore the effects of the lesions on neural activity in the downstream target regions of the prostriata partly because many previous studies showed that specific brain lesions lead to hypoactivity in some closely connected regions (Jenkins et al., 2006; Vann and Albasser, 2009; Dupire et al., 2013). In addition, neural activity in the downstream regions may also be affected by behavioral deficits in lesioned animals.

## Materials and methods

### Animals

Thirty-two adult Sprague-Dawley (SD) rats of both sexes (280–350 g, from the Beijing Vital River Laboratory Animal Technology Co., Ltd.) were used as experimental subjects. The number of male and female rats used in this study was not recorded. All the rats were kept in the same room with standard laboratory conditions (12 h light/dark cycle; setting temperature = 22 ± 2°C; setting humidity = 50 ± 10%), as well as free access to food and water in this present study. All experimental procedures were followed in accordance with the protocols that have been approved by the Institutional Animal Care and Use Committee of Guangzhou Medical University.

### Animal surgery

All animals were randomly divided into control group (no injection; *n* = 10), sham group (the prostriata was injected with 0.9% sterile saline; *n* = 10)and experimental group (the prostriata was injected with 10 mg/ml Ibotenic acid; *n* = 12). The rats were anesthetized with sodium pentobarbital (40 mg/kg, i.p.) before the operation. After the rats were completely unconscious, the rats were placed in a stereotaxic frame. After top-hair shaving and disinfection, a 2-cm midline incision was made on the top of the rats’ head and the nose clip was adjusted to make the bregma and lambda at the same level. The specific location and layers of the prostriata in the rats have been recently identified (Chen et al., 2020; Lu et al., 2020). A suitable drill was used to make two holes (one per side) in skull over the target area and a 0.5 μl Hamilton syringe was used to deliver the injections (0.3 μl per side; each for 10 mins). The coordinates for all the prostriata injections are -8.72 (bregma), 3.20 (off midline), and 4.05 (depth). After the injections, the syringe was kept in place for another 10 mins and then slowly pulled out. After the wound suturing the rats were placed on a warm electric blanket until they woke before returning them to their cages. Two rats in the sham group did not survive on the surgery day likely due to some issue with the anaesthetization.

### Behavioral tests

After 10 days of postoperative recovery, the rats were brought to the behavioral testing room approximately 2 h before the tests. During the next 8 days, the rats were subjected to behavioral tests, including the open-field test (OFT; on the 11th day), elevated plus-maze (EPM; on the 12th day) test and Morris water maze (MWM; on the 13th–17th days) test. On the 18th day, the platform was removed from the pool and each rat was tested for 2 min to measure spatial learning and memory capacity. All the rats were sacrificed using the same procedure (see Brain tissue preparation below) immediately after the last behavioral test.

#### Open-field test

The apparatus with a volume of 100 cm × 100 cm × 40 cm (length × width × height) was evenly divided into 25 squares (20 cm × 20 cm). The 25 identical squares were marked as 1–25 from right to left starting from the bottom right corner, of which squares 7, 8, 9, 12, 13, 14, 17, 18, and 19 were set as the area of center (Hu et al., 2017). In a dark, quiet and well-ventilated room, the rats were placed in order from area 1 head down and allowed to explore for 5 mins. After each test, the apparatus was cleaned and disinfected with 75% alcohol before testing the next rat. The whole experiments were recorded by video, and the related software was used for data analysis at the end of the experiments.

#### Elevated plus-maze test

The apparatus consists of two open arms (length × width = 50 cm × 10 cm), two relatively closed arms (length × width × height = 50 cm × 10 cm × 40 cm) and a central platform (length × width = 10 cm × 10 cm) connected to four arms. The open arms, closed arms and area of center were all black and were perpendicular to each other (Bruijnzeel et al., 2019; Knight et al., 2021). The plus maze was fixed on a cross bracket with the same length as its arms, and the cross bracket was 50 cm above the floor. In a dark, quiet and well-ventilated room, the rats were placed in order from the central area head down and allowed to explore for 5 mins. The staying time of the rats in the open arms, the closed arms and the central area as well as the number of times the rats entered the open arms were recorded. After each test, the feces were removed after each trial and disinfected with 75% alcohol.

#### Morris water maze test

The Morris Water Maze (MWM) (120 cm in diameter, 50 cm in height) was placed in a quiet and well-lit room (Warner et al., 2013). The inner wall of the MWM was painted black and has four typical patterns on it, including triangles, crosses, circles, and squares, to help the rats remember the position of the platform. The circular pool was divided into four quadrants and a circular platform with a diameter of 11 cm was placed in the center of the first quadrant (between the triangle and square), and then 25 cm-deep water was poured into it (the height of the water surface just immersed the platform 2–3 cm; water temperature = 22 ± 2°C). A high-definition camera was placed on the top of the MWM to record the trajectories of the rats, which were analyzed with the EthoVision XT 14 system. Since the rats tended to stay in a dry environment and hated staying in water, they would rush to find the platform to stay away from the water. For the next 6 days, the rats were trained to find the platform, but on the sixth day the platform was removed from the pool to test animals’ memory of the platform (Sun et al., 2021). In the first 5 days, the rats were tested four times a day, each time they were put into the pool from the center of a different quadrant and each rat was gently released along the maze wall into the pool in the four quadrants every day (Table 1). The rats were allowed to train for a maximum of 2 mins each time (Vorhees and Williams, 2006). If the rat found the platform within 2 mins, it was allowed to stay on platform for 3 s and then take it out. If the rat could not find the platform in 2 mins, the rat would be guided to find the platform and allowed to stay on the platform for 10 s. After each rat completed the training, the rat was wiped dry and placed in a cage with suitable temperature to rest for 20 mins (Zhang et al., 2015). On the sixth day, the platform in the first quadrant was removed from the pool, and each rat was placed in the pool from the center of the third quadrant and explored for 2 mins. We chose the third quadrant, the farthest from the platform, as the quadrant to release the rats into the pool, to reduce the possibility for the rats to find the platform location by chance.

**TABLE 1:** Summary of the starting position of each training (hidden platform located at NE).

Day	Trial 1	Trial 2	Trial 3	Trial 4
1	SW	SE	NW	NE
2	SE	NE	SW	NW
3	NW	SW	NE	SE
4	NE	NW	SE	SW
5	SW	NW	SE	NE
6 (Probe)	SW			

### Brain tissue preparation

After completing a series of behavioral experiments, the rats were deeply anesthetized with an overdose of sodium pentobarbital (60 mg/kg, i.p) until they were completely unconscious and then perfused transcardially with 0.1 M phosphate buffer (PB, PH = 7.3) followed by 4% paraformaldehyde (PFA) in chilled PB. After the rat’s liver turned white and hardened, the brain was taken out and postfixed in 4% PFA at 4°C overnight. For the next 3–4 days, the brain was stored in 15 and 30% sucrose, in sequence, until the brain sank to the bottom of the bottle. The brain was cut into the left and right hemispheres along the midline, and then the hemispheres were sectioned into 40 μm thick sequential sagittal sections with a freezing microtome (Leica CM3050 S). The brain sections were stored in cryoprotectant for subsequent experiments (Chen et al., 2020; Lu et al., 2020).

### Nissl stain

The sections containing the prostriata were selected from the cryopreservation solution and rinsed with PB. The sections were then mounted on slides and dried in an oven at 37°C. For Nissl staining, the sections were placed in xylene and gradient alcohol (100, 95, 85, and 70%) for 5 mins each and then stained in 0.1% Cresyl Violet solution for 20 mins and immersed in distilled water for 5 mins. Finally, the sections were dehydrated in 85, 95, and 100% ethanol for 5 mins each before being placed in xylene (two times) and coverslipped.

### Immunohistochemistry

The sagittal sections containing the major downstream target regions of the rat prostriata were selected for c-fos IHC. These regions include the PrSd-PoS, LD, LP-Pul, PTN, and VLG and are directly innervated by the prostriata (Chen et al., 2021). In addition, we also evaluated c-fos expression in zona incerta (ZI) and substantia nigra (SN), which are not the targets of the prostriata. Selected sections were rinsed three times with 0.1 M PB and immersed in 0.3% hydrogen peroxide for 10 mins. Then the sections were rinsed three times again and blocked in 5% BSA for 60 mins. At the end, the sections were incubated with primary antibody against c-fos (200 μg/ml, 1:200, Boster Biological Technology, Wuhan, China) diluted with the 0.1 M PB overnight at 4°C. On the next day, the sections were incubated in the secondary antibodies (biotinylated goat anti-mouse/rabbit IgG, Boster) for 60 mins after thorough rinse with 0.1 M PB. The sections were then rinsed again and immersed in the Streptavidin-Biotin Complex solution (SABC kit, Boster Biological Technology) for 60 mins. After rinse with 0.1 M PB, the sections were incubated in 3, 3-diaminobenzidine (DAB) solution for 3 mins in a dark environment. Finally, the sections were mounted on the slides, dehydrated and coverslipped.

### Image capture and data analysis

All behavioral tests, including OFT, EPM, and MWM, were recorded and collected by the EthoVision XT 14 system. The movement trajectories of the rats were recorded by high-definition cameras. Statistical analyses were performed using the IBM SPSS 20.0 software. The performance of the animals during OFT, EPM, and MWM was analyzed using ANOVAs. The results in the graphs were presented by mean values ± standard deviations (mean ± SD). In all statistical analyses, *p* < 0.05 was considered significant. The Nissl- and IHC-stained sections were digitized with a scanner (Aperio CS2, Leica). Cell counts of c-fos positive neurons (for neuronal activity) in selected brain regions (i.e., PrSd-PoS, LD, LP-Pul, PTN, VLG, ZI, and SN) was performed using image J software and the statistics was done using one-way ANOVA.

## Results

### Localization of the lesions in the prostriata

**FIGURE 1:**
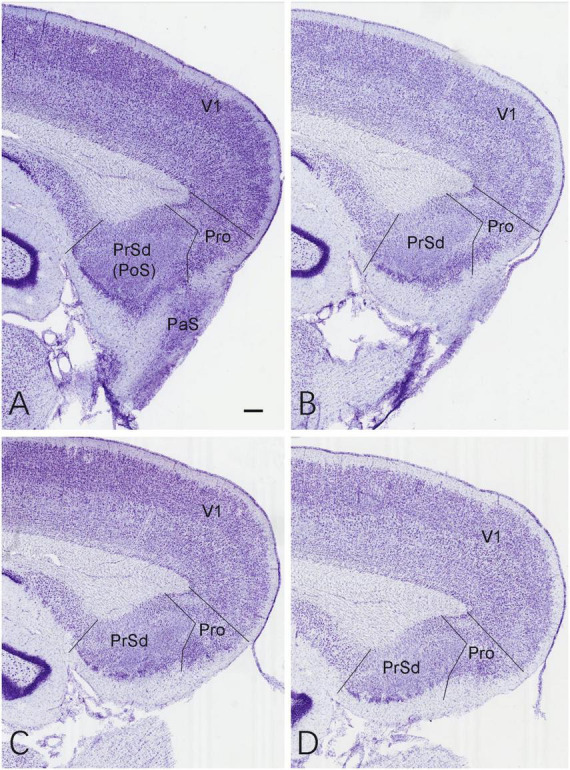
Location and normal cytoarchitecture of the rat prostriata. **(A–D)** Sequential Nissl-stained sagittal sections from the lateral **(A)** to medial **(D)** levels of the prostriata and adjoining regions in a normal (control) rat. Bar in panel **(A)**: 200 μm for all panels.

The location and extent of the prostriata in rats have been demonstrated recently in both Nissl and calbindin-D28k (CB) stained sections (Lu et al., 2020; Chen et al., 2021). As reported (Lu et al., 2020), the prostriata is a limbic cortex lacking granular layer 4 and having relatively larger cells in its superficial layers 2–3 compared to adjoining regions and is located at the junction among the PrSd-PoS, RS, parasubiculum (PaS), and the medial visual cortex (see Figure 1).

**FIGURE 2:**
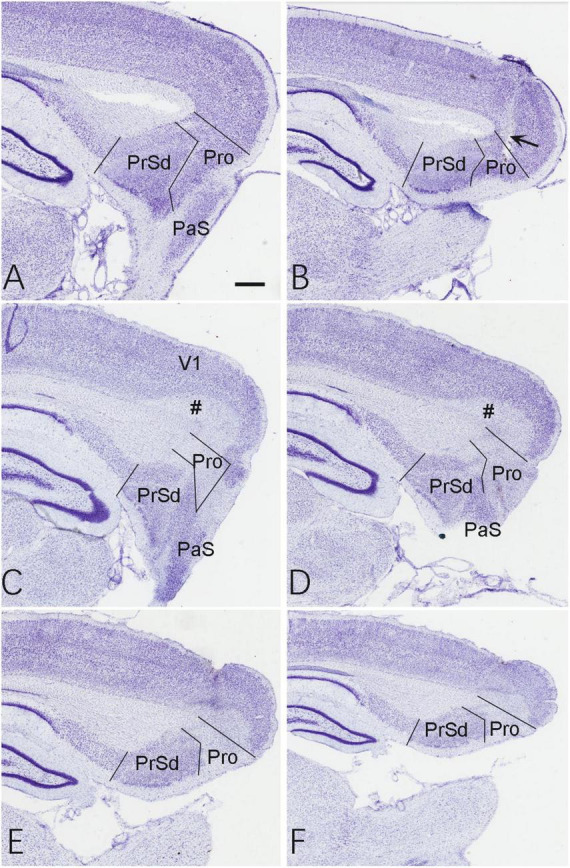
Evaluation of the lesion and its extent. **(A,B)** Two Nissl-stained sagittal sections from the lateral **(A)** and middle **(B)** levels of the prostriata and adjoining regions in the sham group. The arrow in panel **(B)** indicates a needle track of the injection. **(C–F)** Sequential Nissl-stained sagittal sections from lateral **(C)** to medial **(F)** levels of the prostriata and adjoining regions in the experiment group. The #s in panels **(C,D)** indicate layer 6 of the V1. Bar in panel **(A)**: 400 μm for all panels.

Based on the location of the prostriata (Lu et al., 2020; Chen et al., 2021), the extent of the chemical lesions caused by ibotenic acid was evaluated on Nissl-stained sagittal sections of both hemispheres from sham and experimental groups. Compared to the control group (Figure 1), no significant cell loss was observed in the prostriata of the sham group (e.g., Figures 2A,B) although a small number of cells were lost along the needle track (indicated by the arrow in Figure 2B). However, in the experimental group, all animals included for data analysis display bilateral lesions in the prostriata and the lesions were severe and many cells in the prostriata were lost, as shown in the sequential sagittal sections from the lateral to medial levels (the damaged prostriata areas appear pale; see the Pro in Figures 2C–F). In addition, a small portion of the PaS and/or layer 6 of the overlying V1 (indicated by the # in Figures 2C,D) also showed some cell loss, but most of the PrSd-PoS and PaS regions were found to be intact (Figures 2C,D). It should be mentioned that two rats (from experimental group) were excluded from data analysis because the injections missed the target and spared over 70% of the prostriata. In other 10 rats of this group, the injections consistently damaged 70–90% of the prostriata of each hemisphere (e.g., Figures 2C–F).

### Behavioral tests

#### Open-field test

**FIGURE 3:**
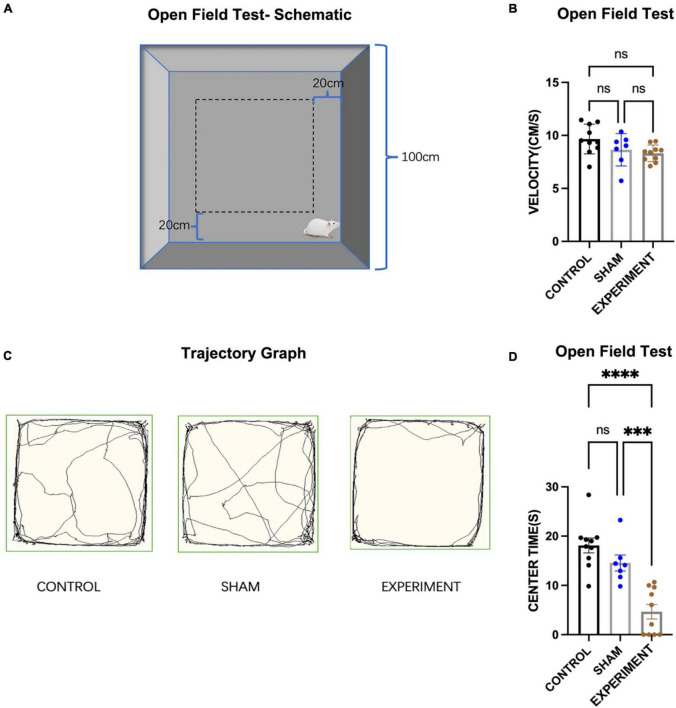
Open-field test. **(A)** Schematic diagram of the open field test. **(B)** The movement speeds of the rats (mean ± SD). **(C)** Movement trajectory paths of the rats. **(D)** The times spent in the central area (mean ± SD). ^***^p < 0.001, ^****^p < 0.0001.

We used the classic open-field test (OFT) (Figure 3A) to assess the locomotor ability of the rats and their interest in exploring space (Hu et al., 2017), and one-way ANOVA for the statistics, which found a significant effect of the group (control, sham and experiment) on time spent in the central area (*F*[2, 24] = 22.47, *p* < 0.0001, [eta-squared: 0.6518]). Post-hoc comparison (Tukey HSD, *a* = 0.05) revealed that the time spent in the central area was significantly reduced in the experimental group compared to both the control and sham groups. Comparison of the control (*N* = 10), sham (*N* = 8) and experiment (*N* = 10) groups showed no significant change in the movement speed (*F*[2, 24] = 3.136, *p* = 0.0617, [eta-squared: 0.2072]). Post-hoc comparison (Tukey HSD, *a* = 0.05) revealed that there is also no significant difference in the movement speed between each two groups (see Figure 3B). Comparison of the movement trajectories (paths) of the control, sham and experiment groups showed that the experiment group were less interested in exploring the center area than the other groups (Figure 3C). Compared with the control group and the sham group, the times spent in the central area was significantly reduced in the experimental group (Figure 3D).

#### Elevated plus-maze test

**FIGURE 4:**
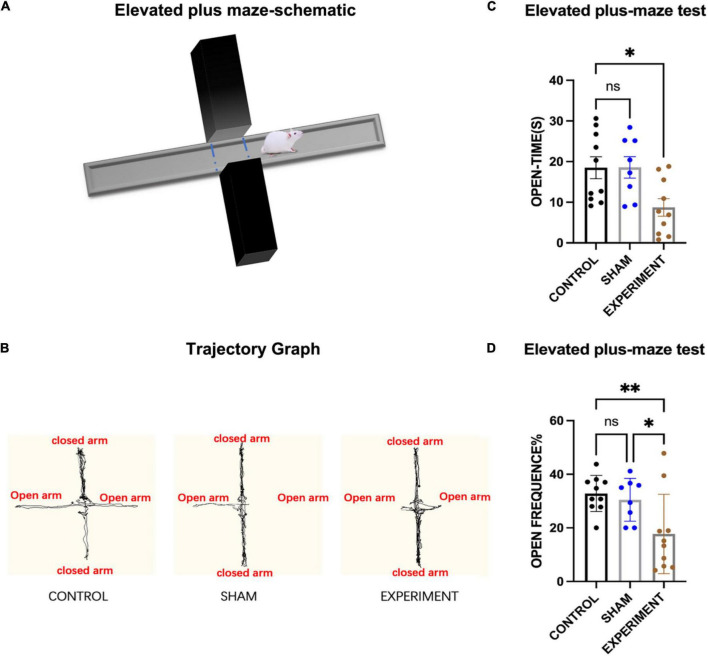
Elevated plus-maze test. **(A)** Schematic diagram of the elevated plus-maze test. **(B)** Movement trajectories of the rats. **(C)** The times spent in the open arms (mean ± SD). **(D)** The frequencies entering the open arms (mean ± SD; open frequency = number of times entering open arms/total number of times entering open and closed arms). *p < 0.05, ^**^p < 0.01.

The elevated plus-maze (EPM) (Figure 4A) was used to assess anxiety of the rats in the control (*N* = 10), sham (*N* = 8), and experiment (*N* = 10) groups, and one-way ANOVA was used for statistics. It was observed that most of the movement trajectories of the rats were in the closed arms (Figure 4B), but the rats in the control and sham groups explored more in the open arms. The times spent in the open arms for the experimental group was significantly reduced compared to the control and sham groups (*F*[2, 25] = 5.30, *p* < 0.0121, [eta-squared: 0.2977]) (Figure 4C). Post-hoc comparison (Tukey HSD, *a* = 0.05) showed no significant changes between the sham and control groups (Figure 4C). As for the frequency of the rats entering the open arms, experimental group entered the open arms less frequently than those in the control or sham groups (*F*[2, 25] = 5.71, *p* = 0.0091, [eta-squared: 0.3136]) (Figure 4D). Post-hoc comparison (Tukey HSD, *a* = 0.05) revealed no significant changes between the sham and control groups (Figure 4D).

#### Morris water maze test

**FIGURE 5:**
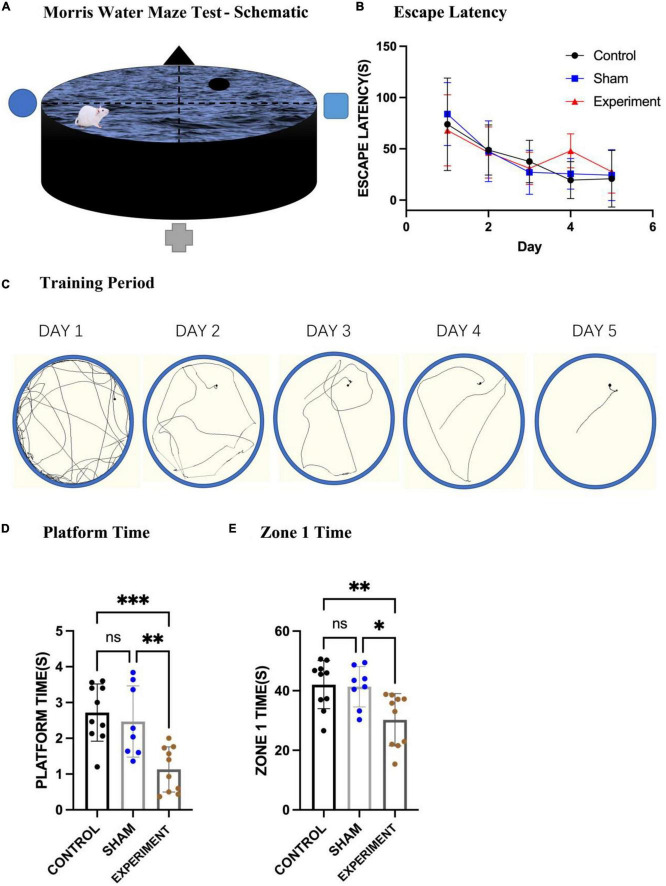
Morris water maze test. **(A)** A schematic of the MWM apparatus. **(B)** The mean times of the three groups swimming to the platform (mean ± SD). **(C)** Changes in locomotion trajectories of the rats reaching to the platform during the 5-day training period. **(D)** The times spent in the original platform area after the platform was removed (mean ± SD). **(E)** The times stayed in zone 1 on the sixth day of the test (mean ± SD). *p < 0.05, ^**^p < 0.01, ^***^p < 0.001.

The Morris water maze (MWM) was used to evaluate spatial learning and memory of the rats (Figure 5A), and one-way ANOVA used for statistics. From the movement trajectory, it was observed that the rats found the platform more quickly after training in the first 5 days (Figure 5C). The average time to reach the platform in the first 5 days in the control (*N* = 10), sham (*N* = 8), and experimental (*N* = 10) groups all decreased with the increase of training time (Figure 5B). After removing the platform on the sixth day the experimental group stayed on the original platform region for less time than the control and sham groups (Figure 5D) (*F*[2, 25] = 11.01, *p* = 0.0004, [eta-squared: 0.4684]). In addition, since the platform was in zone 1, we also calculated the times the rats spent in this zone. The experimental group stayed in zone 1 for less time than the control and sham groups (Figure 5E) (*F*[2, 25] = 6.653, *p* = 0.0048, [eta-squared: 0.3474]). Improvements in escape latency across the 5 days of MWM training were analyzed with a mixed-model ANOVA with Group as a between-subjects factor and Day (1–3) as a between-subjects factor. The statistics shows that Day *F*(3.060, 76.51) = 17.41, *p* < 0.0001; Group *F*(2, 25) = 0.3075, *p* = 0.7380; interaction (group × day) *F*(8, 100) = 1.114, *p* = 0.3601. Although the interaction is not significant and this may suggest that the trend over days did not differ significantly by group, our results from the sixth day (the platform was removed) show that experiment group stayed on the original platform region for less time compared to other two groups (Figure 5D), suggesting at least the trend of the deficits in learning and memory over days. Finally, using the first 2 days as short-term memory practice (Bruszt et al., 2021), we found that the short-term spatial memory in the experimental group was not significantly affected (Figure 5B).

#### C-fos expression in downstream targets of the prostriata

**FIGURE 6:**
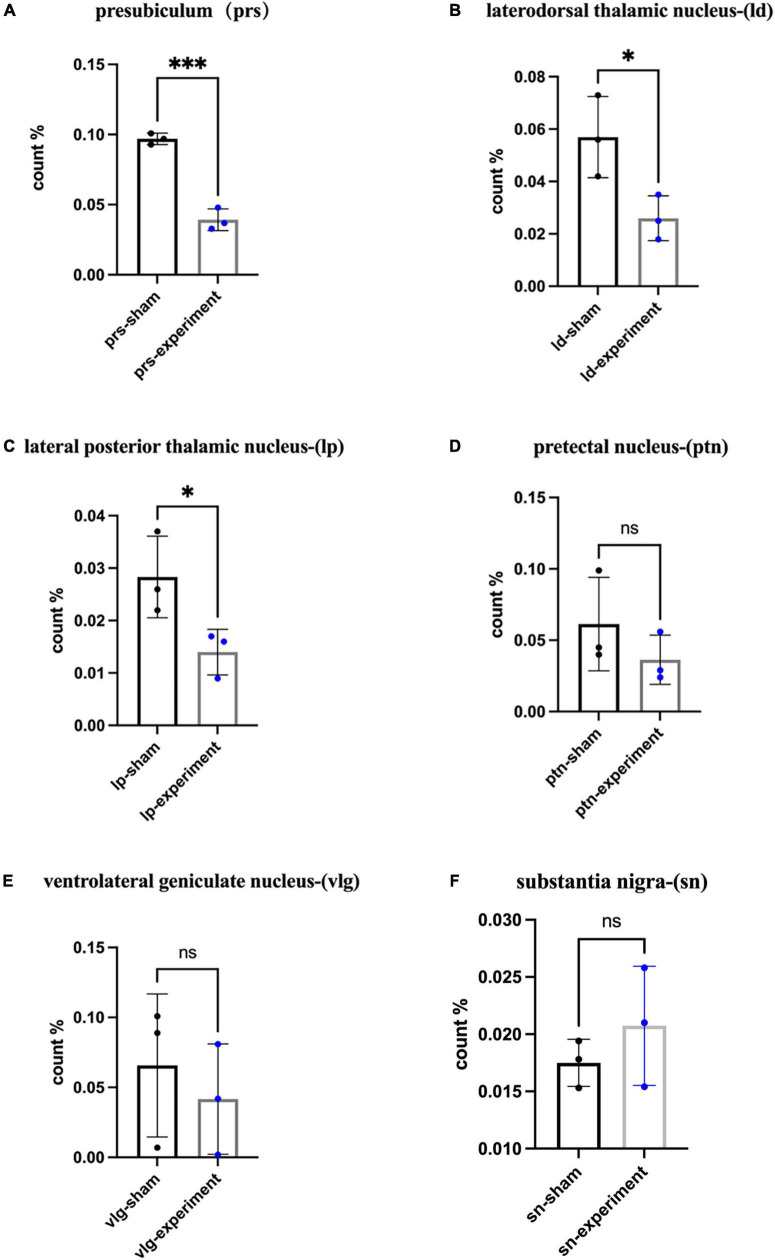
The percentage of c-fos expressing cells in the target areas. **(A)** The ratios of the number of c-fos expressing cells in the PrS-PoS (mean ± SD, t_4_ = 11.43, *p* = 0.0003). **(B)** The ratios of the number of c-fos positive cells in the LD (mean ± SD, t_4_ = 3.03, *p* = 0.0388). **(C)** The ratios of the number of c-fos expressing cells in the LP-Pul (mean ± SD, t_4_ = 2.78, *p* = 0.0494). **(D)** The ratios of the number of c-fos expressing cells in the PTN (mean ± SD, t_4_ = 1.17, *p* = 0.3065). **(E)** The ratios of the number of c-fos expressing cells in the VLG (mean ± SD, t_4_ = 0.64, *p* = 0.5551). **(F)** The ratios of the number of c-fos expressing cells in the SN (mean ± SD, t_4_ = 1.00, *p* = 0.3739) (ratio = the number of c-fos positive neurons/size of area examined for each structure). *p < 0.05, ^***^p < 0.001.

Our previous studies revealed that the major target regions of the prostriata in rats include the PrSd-PoS, LD, LP-Pul, PTN, and VLG (Chen et al., 2021). Many previous investigators used c-fos expression as a tool to indicate activity of activated neurons (Sagar et al., 1988; Dragunow et al., 1989; Herrera and Robertson, 1996; Velazquez et al., 2015). In addition, c-fos expression was also used to indicate hypoactivity in closely connected regions after specific brain lesions and behavioral tests including learning and memory tests (Herrera and Robertson, 1996; Jenkins et al., 2006; Vann and Albasser, 2009; Dupire et al., 2013). Since the rats were sacrificed immediately after the probe trial in the MWM, neural activity in the target regions of the prostriata could be affected by both the behavioral deficits and loss of afferents, which could cause the hypoactivity. Therefore, we examined c-fos expression in the major target regions to evaluate the effects of the prostriata lesions on the target regions. Targeted sections from three rats in each of the sham and experiment groups were randomly selected for c-fos IHC staining, and student *t* test was used for analysis. The results showed that the ratios of the number of c-fos positive neurons in the PrSd-PoS (*P* < 0.01), LD (*P* < 0.05), and LP-Pul (*P* < 0.05) was significantly reduced in experimental group compared to sham group (Figures 6A–C). However, the ratios of the number of c-fos positive neurons in the PTN (*P* > 0.05) and VLG (*P* > 0.05) of the experimental group did not show significant decrease although the ratios tended to decrease (Figures 6D,E). Finally, we also evaluated c-fos positive neurons in the ZI and SN that are not the targets of the prostriata. The ratios of the number of c-fos positive neurons in the ZI and SN of the experimental and sham groups did not show significant changes (e.g., Figure 6F for SN).

## Discussion

The present study has showed that, following the OFT, EPM, and MWM tests, control and sham rats were able to maintain normal anxiety state and use spatial land markers to memorize specific locations. However, the experimental group with bilateral prostriata lesions displayed deficits in spatial learning and memory as well as possible anxiety, compared to control or sham groups. There was not significant effect on motor abilities of the lesion rats. However, we cannot completely rule out the possibility that some vision impairments may occur in some rats with some lesion in layer 6 of the V1 (see “Results” section). V1 is a large region and its layer 6 is mainly the region that initiates feedback projections to the DLG and thus would not significantly affect vision perception. Therefore, we believe that vision impairments, if any, are minimum or not significant in the present study. This conclusion is supported by our findings that the lesion rats did not show significant decrease in moving velocity in the OPT compared to control and sham groups and that the three groups did not show significant difference in escape latency during the first three days of the MWM training. These findings cannot be explained if the lesion rats had significant vision impairments. Taken together, we believe the behavioral changes observed in this study is the results of increased anxiety and deficits in spatial learning and memory.

### Prostriata and spatial learning and memory

In this study, we used the neurotoxin ibotenic acid to damage neurons in the prostriata. The lesion caused by single injection covers most of the prostriata (Figures 2C–F) since the prostriata in rodents is small in size (Lu et al., 2020; Chen et al., 2022). Our results showed that the movement speeds of the rats in the control, sham and experimental groups did not show significant changes, so there was no significant difference in the movement ability among the three groups. We conducted escape training for each group for 5 days and found that the rats in all groups succeeded in finding the platform, suggesting the acquisition of spatial learning by the end of the training period even in the experiment group. However, when we took the first 2 days as short-term memory practice and the last 2 days as long-term memory one (Bruszt et al., 2021), we found that the short-term spatial memory in the experimental group was not significantly affected while the long-term spatial memory ability changed (Figure 5B). These results indicate that it is difficult for the rats in the experimental group to find the platform area on later days even they can find the platform area on the first day. Therefore, it is possible that bilateral damages to the prostriata reduce not only the spatial navigation ability but also their memory ability. We also counted the times the rats stayed in zone 1, where the platform was located and found that the times also decreased for the rats in the experimental group (Figure 5E). This may indicate that the prostriata-lesion rats had poorer ability to recognize precise and imprecise positions. Because the prostriata plays an important role in analysis of information from peripheral visual field (Rockland, 2012; Yu et al., 2012; Mikellidou et al., 2017; Tamietto and Leopold, 2018), the rats in the experimental group are likely not good at using the signs on the walls of the MWM apparatus compared to the other two groups.

### Possible neural mechanisms underlying the spatial learning and memory impairment

Our recent studies have revealed that the prostriata receives direct projections from the visual cortex, AD, AV, LD, RS, Sub, PrSd-PoS, and MEC (Ding, 2013; Ding et al., 2020; Hu et al., 2020; Lu et al., 2020; Chen et al., 2021). All these structures are important components of spatial memory processing system and the AD and PrSd-PoS contain many head direction cells (Cho and Sharp, 2001; Hafting et al., 2005; Taube, 2007; Tsanov et al., 2011). It is likely that damage to the prostriata would impair the processing of spatial information participated by these structures such as landmark signal integration and accurate visual navigation.

On the other hand, the prostriata has strong projections to the PrS-PoS (Chen et al., 2021), which heavily innervates the MEC. The MEC contains many head direction cells, place cells and grid cells with the latter two function as grasping the location and integrating spatial information, respectively (Taube, 2007; Moser et al., 2008). Place cells are usually believed to help animals reach the target position by comparing the similarity between the current position and the target position (Burgess and O’Keefe, 1996; Bush et al., 2015). Grid cells are thought to integrate both location and direction information and to provide a path integration input to place cells (Hafting et al., 2005; O’Keefe and Burgess, 2005; McNaughton et al., 2006; Rolls et al., 2006; Solstad et al., 2006; Taube, 2007). Therefore, it is possible that the prostriata may function as a node integrating visual landmark information, head direction and position information. When the prostriata is damaged, the functions of head direction cells, place cells and grid cells in the downstream target regions such as the PrSd-PoS, LD, and MEC would be impaired. This appears reflected in the reduction of c-fos positive neurons in some of the downstream structures such as the PrSd-PoS and LD.

### Prostriata and anxiety

According to the results of the OFT, the times spent in the central area for the lesion group was significantly reduced in comparison with the other groups. According to this result, we could infer that the rats in the experiment group had decreased interest and even feared in the exploration of a novel environment (Hu et al., 2017). Our recent studies in rodents have found that the prostriata connects to the LP-Pul and ORBm (Hu et al., 2020; Chen et al., 2021). The LP-Pul, which receives strong and direct projections from the prostriata, were reported to be important in fear processing and in activating stress responses via its connections with amygdala (Goosens and Maren, 2001; Herman et al., 2005; Arend et al., 2008; McFadyen, 2019). The ORBm also plays an important role in emotional changes, including reward, aggression, and aversion (Butter et al., 1970; Butter and Snyder, 1972; Rolls, 2019). Therefore, we speculate that the rats with prostriata lesion may be less interested in exploring the environment and display increased anxiety due to the impairment of the prostriata and reduction of the afferents to the LP-Pul and ORBm. In addition, the prostriata belongs to and connects heavily with the limbic system, whose impairment could also increase anxiety and fear (Meyer et al., 2012; Deal et al., 2016). The possible fear and anxiety of the experimental group were also reflected in the EPM test. In this study we found that the times and frequencies of the lesion rats entering the open arms were less than those in the other two groups. Due to anxiety and/or lack of interest, the rats in the experimental group could be reluctant to explore relatively open area in both the OPT and EPM tests. In addition to possible emotional changes affecting the behavioral performance of the rats, we cannot ignore the impact of the reduced ability of the lesion rats to analyze the information from the peripheral visual field. Since the prostriata plays an important role in analyzing information from the peripheral visual field, the ability of the rats with damaged prostriata to explore in a distant unfamiliar environment would also be reduced (Yu et al., 2012; Mikellidou et al., 2017; Tamietto and Leopold, 2018; Lu et al., 2020; Chen et al., 2021). Therefore, we speculate that the changes in the behavioral performance of the rats following bilateral prostriata lesions could be the result of the simultaneous effects on the emotional and visual abilities of the rats.

## Data availability statement

The original contributions presented in this study are included in the article/supplementary material, further inquiries can be directed to the corresponding author.

## Ethics statement

This animal study was reviewed and approved by Institutional Animal Care and Use Committee of Guangzhou Medical University.

## Author contributions

S-LD: conceptualization. S-YZ, J-YZ, C-HC, X-JX, and H-RC: data generation. S-YZ and S-LD: data analysis and manuscript writing. S-QC and SL-D: supervision. All authors have read and approved the submitted manuscript.

## Abbreviations

A2: association auditory cortex
AD: anterodorsal thalamic nucleus
AM: anteromedial thalamic nucleus
ATN: anterior thalamic nucleus
AV: anteroventral thalamic nucleus
DLG: dorsal lateral geniculate nucleus
LD: laterodorsal thalamic nucleus
LP-Pul: lateroposterior thalamic nucleus-pulvinar complex
MEC: medial entorhinal cortex
NHP: non-human primates
ORBm: medial orbitofrontal cortex
PaS: parasubiculum
PN: pontine nucleus
PoS: postsubiculum
PrS: presubiculum
PTN: pretectal nucleus
RS: retrosplenial cortex
SN: substantia nigra
Sub: subiculum
V1: primary visual cortex
VLG: ventral lateral geniculate nucleus
ZI: zone incerta.
